# Thrombus can enhance on delayed enhancement imaging

**DOI:** 10.1186/1532-429X-16-S1-P97

**Published:** 2014-01-16

**Authors:** Roja Tumma, Scott Feitell, Yuchi Han, Harold Litt

**Affiliations:** 1 Radiology and imaging, Hospital of University of Pennslyvania, Philadelphia, Pennsylvania, USA; 2 Cardiology, Drexel University College of Medicine, Philadelphia, Pennsylvania, USA; 3 Cardiology, Hospital of University of Pennsylvania, Philadelphia, Pennsylvania, USA

## Background

To examine the diagnostic accuracy of cardiac magnetic resonance imaging (CMR) in differentiating thrombus from myxoma Thrombus is thought not to enhance on CMR, myxomas are thought to show mild heterogenous enhancement on delayed imaging. It is sometimes difficult to differentiate them, and this may lead to misdiagnosis, which would significantly impact treatment. Thrombi can have varying signal intensities depending on their age and fibrous composition in T1 and T2 imaging.

## Methods

A retrospective review of CMR performed from January 2000 to June 2013 was conducted with waiver of consent which was approved by our institutional review board. Analysis of a total of 46 masses diagnosed as myxoma or thrombus on CMR were compared with histopathology reports or follow up imaging to determine the diagnostic accuracy of CMR. All patients underwent CMR on a 1.5 Tesla scanner with EKG gating following the same protocol.

## Results

**Figure 1:**
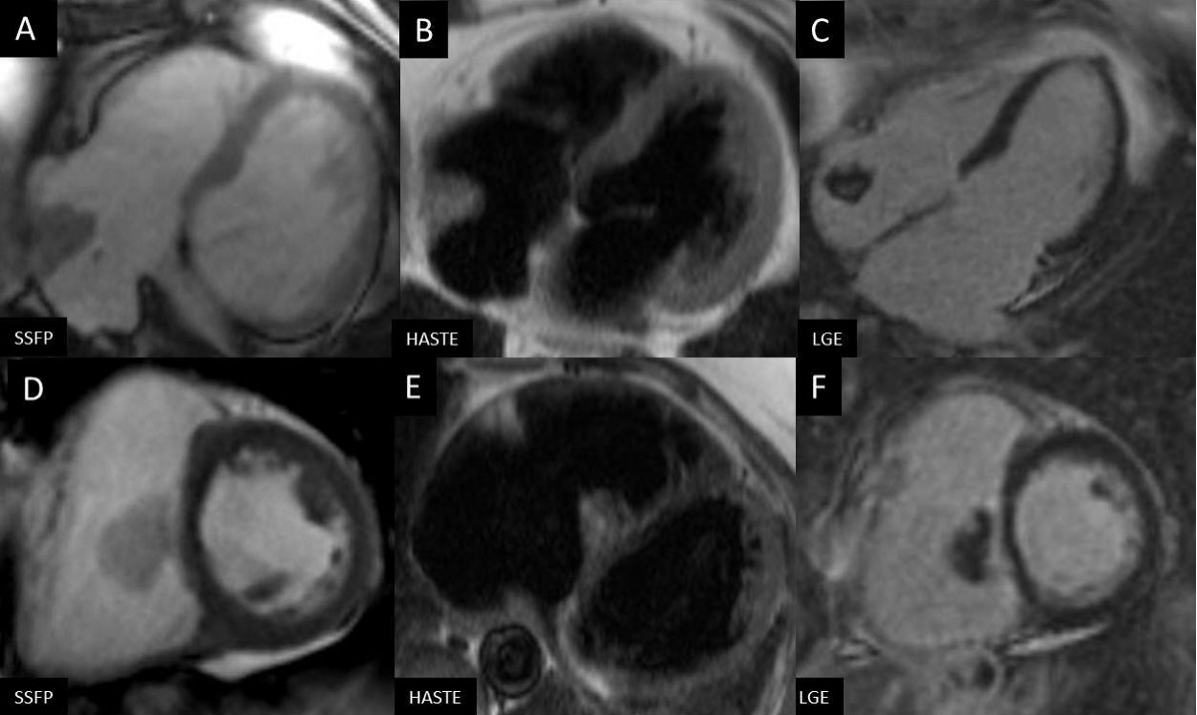
**Images A-C: CMR demonstrating a mass with mild delayed enhancement suggestive of myxoma, later proven to be thrombus; Images D-F: CMR demonstrating a mass diagnosed as myxoma, confirmed pathologically**.

Of the 46 masses reviewed, sixteen masses were diagnosed as myxoma on CMR, while post-operative pathology reports revealed 11 of these to be myxomas, 4 were found to be thrombi, and 1 was an artifact associated with mitral valve prosthesis. Of the 30 patients that were diagnosed with thrombus, all were placed on anticoagulation and monitored closely. One patient demonstrated no resolution of mass at time of follow up, and subsequently underwent surgery which revealed a myxoma. Of the 4 misdiagnosed myxoma cases, all had mild patchy delayed enhancement on CMR imaging making differentiation difficult (Figure 1). Of the one misdiagnosed thrombus case, the mass was very small and could only be seen as a non-enhancing defect in contrast to the high signal seen on first-pass. The positive predictive value for myxoma and thrombus diagnosis by CMR is 69% and 97%, respectively. Overall, CMR was able to correctly differentiate between thrombus and myxoma in 87% of cases.

## Conclusions

Delayed enhancement is one of the important factors distinguishing a myxoma from a thrombus. However we found in our study that some thrombi may show mild delayed enhancement because of varying tissue composition and result in a misdiagnosis. CMR evaluation can also be difficult in the presence of artifacts (motion, valve prosthesis, intracardiac leads) and arrhythmias. Thrombi may show patchy enhancement on delayed imaging and can be confused as atrial myxoma on CMR.

## Funding

None.

